# East and West

**Published:** 1887-10-15

**Authors:** Leslie Keith

**Affiliations:** Author of "The Chilcotes," "Uncle Bob's Niece," etc.


### East and West.

By Leslie Keith,

Author of "The Chilcotes," "Uncle Bob's Niece," etc.

(*Continued from p. 32.*)

### Chapter III.—Lavender Lane.

David and Mary Jardine were not allowed to go forth on their adventures without a liberal equipment of advice. That none of the advisers had any personal experience to guide their counsels did not matter in the least. In this free country everybody has a right to an opinion. It is a well-known fact that the subtlest critics and the smartest reviewers are those who can neither paint the pictures nor write the books it is their mission to cut into mincemeat for the amusement of the public; and if we were all to shut our mouths till we had mastered our subject, there would be no such thing as conversation or freedom of discussion. Therefore, those honest folks had every right to speak with authority when they brought their wisdom to Mary. What was London to them? A bigger Edinburgh—a possibly wealthier Glasgow—a far-off place where people went and did not return, presumably because they grew rich and proud; or if by chance they did revisit the old home, it was to come back changed, speaking a "clippet" English, and with new views as to victuals.

This same matter of food exercised the local mind considerably; and Mary's modest luggage was, in the end, swelled to twice its original proportions with specimens of native produce—a bag of oatmeal, a home-cured ham, eggs, butter, scones, a skim-milk cheese. If she and David had been going into exile at the North Pole they could scarcely have been better provisioned.

On the question of the oatmeal, this essential in the manufacture of porridge, the public view was unanimous. "You'll no' let David fa' into new-fangled ways; you'll see that he sups them every morning." In Logan-lea the national dish is always mentioned in the plural. To this, and much else that need not be recorded, Mary gave a willing assent. Old custom, old habit - she was not the less likely to cling to these, where such faithfulness was possible, because everything else about her was sure to come with the force of a novelty. As a further help to the wanderers, there was disinterred from the general memory the name and address of a former dweller in Logan-lea, a certain Mrs. Carter, who had married a Southerner, and was supposed to have done exceedingly well in the provision line. With a letter, written by the minister himself, commending the pair to her graces, and with that other important document confided to her care by the schoolmaster, Mary felt herself armed against all emergencies.

Often and often on the long and weary journey did she pull out the letters and look at them secretly. The one was addressed to Park Lane and the other to Lavender Lane, Bethnal Green. For all her ignorance could tell, the one place might rub shoulders with the other. Lavender Lane pleased her ear the better of the two. It had a homely sound; it spoke of modest country delights, of green trees and flowers that come with the summer, and fields not far off, perhaps. London ceased to be a terror when she looked at this name—if she could have a tree or two and some grass, and perhaps a distant vision of hills—poor Mary, how little she knew!—she could face everything else: the parting with the old and the hazard of the new. As for David, he wanted nothing but streets—streets full of men and women, who took life at its fullest beat; full of shops where books were sold; where he should one day see his own books occupying a foremost place; where publishers were eagerly waiting for the new poet's advent, and ready to outbid each other for his wares. David's London was, it will be seen, even a more impossible place than Mary's.

The real London swallowed them up at last. It was nightfall when they arrived; a spring night, but the lamps had already routed the short-lived twilight, though for hours to come the endless procession with its endless roar would throng the thoroughfares.

When they stood on the platform, their boxes and bundles piled round them, Mary's courage suddenly forsook her. "Oh, David!" she said, clinging to his arm.

"Don't be frightened. I'll take care of you, Mary," said David, his eyes bright, his head erect. The confusion of hurrying porters, of distracted travellers, the shriek of arriving and departing engines, all these breathed of life, and were as wine to him.

Nevertheless, David did nothing but stand still and look about him, which scarcely mended matters; and when at last—their fellow-travellers having been whisked away in cabs and hansoms—a porter at leisure took pity on their helplessness, it was Mary who pulled out the letter and explained that they wished to make for the address written there.

"Lavender Lane, Bethnal Green," read the porter, giving the words a new sound to her ear. He scratched his head, and mentioned various routes by which that outwork of the east might be reached. It sounded to the poor girl as complicated as a direction to the moon, and yet she had supposed herself to be in London.

"Is it a long way?" she faltered.

"It's a goodish bit," he answered, with the vagueness that characterises his kind when taken off the accustomed beat. But David now cut the Gordian knot by saying decidedly—

"Let us take a cab."

"Four-wheeler or 'ansom, sir?"

"Four-wheeler," said David, boldly, at a hazard.

So David and Mary had their first experience of a London "growler," and a very narrow little box they found it. Mary's attention was divided between the arrest of the parcels, that kept sliding with an almost human perversity from the narrow seat in front of them, and an innocent fear lest the cabman might be taking them the wrong way. The London cabby seldom fails to know his way through the great maze, though he sometimes leads the guileless stranger by a circuitous route to his destination. From King's Cross to Bethnal Green is, however, a tolerable distance, and if—when your passenger happens to be a young man from the country—well, if the fare happens to be tolerable too—what then? The young man from the country paid no heed to the parcels that fell on his shins; his thoughts and his glances alike were fixed on the wonderful panorama of the streets. The endless tramp of feet, the endless passing of faces—seen but to vanish; hundreds, thousands, tens of thousands of them—whence did they all come, whither were they going? It seemed as if no city could be big enough to hold all those multitudes. Streets, nothing but streets; streets where the gaslights burned decorously, and streets where it flared and flickered over little shops with bits of meat and fish, and green stuff, ticketed at modest prices. In the latter streets the carriages, cabs, and waggons which had blocked their outsetting were absent, and the carts of the coster took their place. The women hung about them and about the little stalls, eyeing the bits of meat calculatingly, while the men lounged and smoked. There was more leisure here, but there was also more of grimness and ugliness and sadness. Mary felt the sadness sink into her soul. She had scarcely seen one pretty face, and not one that was cheerful and merry with the cheerfulness of country girls; the children had no roses in their cheeks, and the women, too, were sallow and pinched, and looked as if they had been born old.

"Oh, David," said Mary, laying her hand on his, "shall we ever come to the country? Surely it is a long way off?"

"The country!" He turned and looked at her, amazed.

"To Lavender Lane, where Mrs. Carter lives."

"It isn't the country," he said. "If it is," he qualified the assertion, "you don't get me to stop in it even for a night; we've left all that behind us."

Left the country behind them! Was there any country here to leave? It was but a corner of London they traversed, and yet Nature seemed to have fled in horror from the shameful encroachments of man. Mary shut her eyes and tried to banish the endless march of the streets, to think of the silence and calm of her own green hills, but it would not do. The mighty voice of London was in her ears, she had to listen; the faces of London looked into hers, she had to see; her brain throbbed and whirled, she felt that she was being suffocated, stifled, hemmed in with unscalable walls. She had almost ceased to hope or even to wish; she gave up the green now; she even surrendered Lavender Lane. Lane must mean something different from her understanding of it; it was a mockery to suppose it a place of birds, and grass, and shade of trees.

It was, in effect, a little street—a street of the meaner, and yet not of the meanest order, where there are shops, and lodgings for single men; where mangling is done and the humbler sorts of dressmaking; where family life is mostly conducted without a too strict privacy, so that the passing stranger, if he chooses, may liberally share in it, as he shares, whether he will or no, in the savour of frying that assails his nostrils. Perhaps the strongest impression a street of this kind makes is this same odour of cooking—absent from the poorer ones, since, as a rule, in them there is nothing to cook—this hint of the ghosts of last year's dinners and suppers still lingering in the air, divides one's wonder with the troops and bands of children who camp on the doorsteps, and play and quarrel with an awful frankness, and with manners that lack repose.

"Here we are!" cried David, excitedly, as the cab swung round and came to anchorage before a trim little shop with a row of gaslights showing off its contents.

Mary looked up, and reading the name of Carter above the door, she began to pick up and count her stray parcels. She might falter and lose courage by the way, but when it came to doing anything, she was found ready.

A cab with luggage in that neighbourhood is an event: it divides public interest with a hearse, or an accident, or a Salvation Army onslaught. The children, moved by one impulse, immediately deserted the doorsteps; big girls dragging little ones, smaller girls over-weighted with heavy babies, who ought all of them to have been in bed and asleep hours before. All this little crew, and many older folks afflicted with the same curiosity, flocked to the cab, and stood looking at it with that stolid stare that is a distinctly British prerogative. Anything, or nothing, will call it out—chiefly nothing. The other day the writer threaded his way through quite a dense throng of citizens, not a smiling face among them. They were all gazing at a little flock of ducks waddling towards slaughter at a poulterer's. Mary and David, with their air of the provinces and their many odd parcels and bundles, presented a richer field for speculation than the ducks, about whose destiny there could be no mistake.

But what was this pair doing here? what did they want? why had they come? and how long did they mean to remain? Probably Mrs. Carter asked herself all these questions in the short space of time it took her to advance from behind the counter and reach the pavement. She was a buxom woman in the dress of a widow who has had time to conquer an unreasonable grief, and can once more take a pleasurable interest in caps; and that she was a person of authority was evident from the speed with which the crowd parted at her approach.

"Now, now, what's all this?" she said. "What are you standing here for, blocking up the way to the shop?—and a Saturday night, too, when custom is brisk."

The children made a lane for her, and she now faced the travellers. David moved a pace forward.

"Mrs. Carter?" he asked.

"That's my name," she admitted in a neutral tone, as if she wished the crowd to understand that the concession did not commit her to anything.

"You don't remember us," David went on, feeling words to be a little difficult with so many auditors; "we were children when you left Logan-lea—"

"Bless me! have you come from there?" Mrs. Carter's eyes from behind the spectacles travelled inquiringly over the pair. Apparently her scrutiny satisfied her, for she added quickly, "Come in; you can't stand talking in the street. If you're from the North, you're welcome."

With a sweep of her arm she again scattered the advancing bands, drawn near to lose no accent of this dialogue. She seized half-a-dozen of Mary's parcels, and with a "Now children, be off with you!" she led the way into the shop, and passing through it, ushered her visitors into the little parlour behind it.

"Wait here a minute," she said, depositing her share of the travellers' impedimenta on the floor. "Saturday night's a throng night," she explained; and then she smiled. "The old words come back at sight of a Scotch face," she said. They heard her presently giving orders that Mary's trunk should be carried in, and after some further directions to her assistants, she came back and shut the parlour door.

"Now then, tell me who you are," she said.

"I am David Jardine, and this is Mary, my sister."

"Bless me!" said Mrs. Carter again, but the exclamation was this time tinctured with respect. There had been Jardines in Logan-lea almost since there were hills.

"I have a letter," said Mary, producing the precious passport—"a letter from the minister."

Mrs. Carter received and read it in silence. The brother and sister watched her with anxious faces as she broke the seal and travelled slowly over the written lines. She read it twice, being a practical business woman, and then she folded it and looked at David. She looked at him as if he were some abnormal specimen of natural history—a young man who had come to live in London by making poetry.

She knew nothing of that manufacture. People did not come to Lavender Lane to buy it; they only bought groceries for the vile body. Being a wise person, however, she maintained an abeyant mood, and contented herself with asking some practical questions.

"How did you come?"

"By train—to King's Cross."

"And from there?"

"We took a cab," said David, his pride in his quickness of resource speedily damped by her disapproving shake of the head.

"What did you pay the man?" was her next demand. David named the sum, and this time she threw up her hands in horror.

"It's little over two miles," she said; "it's clear you're just a couple of babies."

"We didn't know," said Mary, gently, for David's brow was darkening. "It is all so new to us, and it seemed much more than two miles."

Mrs. Carter's face softened as she looked into the girl's tired, sad eyes. "And what are you going to do?" she asked.

"We have some money," said David, with a hint of hurt pride. "If you can tell us where we can get a lodging, we won't trouble you any longer."

"You can stay here for a day or two and welcome, till you look about you," she said. And if her cordiality was a little stimulated by David's statement, who shall blame her? The poor relation is no more welcome in the East than in the West, and in Bethnal Green as in Belgravia, people are the more respected the more they possess.

"I'm keen to hear all the news of the old place, but we'll not talk to-night," she said considerately, looking again at Mary's weary face. "I'll have to go back to the shop, but I'll send you in a bit of supper, and you'll eat it while Emma is sheeting the beds. The rooms are empty, for a mercy—the lodgers only left last week—and though they're but small, they're clean, that I will say for them."

Mary's one desire was to lay her head on the pillow and forget. But she was too excited or too tired to sleep. The small hot room under the roof seemed to stifle her; the procession of the streets passed once again before her weary brain; and the voices from without that invaded the room, the laughter and quarrelling, the Saturday night's revel, accentuated it all and kept the picture vivid. She rose and, partially dressing, sat down by the window to try and secure a little air. Her sad reverie was presently broken in upon by a knock at the door. She rose to open it, dimly recognising that it was David who stood without.

"Mary," he said, "are you sure my papers are all right?"

"Quite sure, David."

"You've looked since we came?"

"Yes; it was the first thing I did."

"Well," he said, "I'll want them on Monday. "I'll take them to a publisher the first thing in the morning, in case anything should happen to them here. There's always a double risk of fire in a shop. We're very snug here, aren't we, and lucky to get settled so soon, though it's a trifle stuffy. You like it, don't you?"

"Yes," she said faintly, "if you do."

"Of course I do! Though it's only the beginning, you know, the first step. I'll soon be able to set you up better than this. You are sure you are not too tired?"

"No, dear, I'm not too tired," she said. She put her arms round him in the darkness and kissed him.

Mary's London was already a lost illusion, a dream that has vanished with the first streak of daylight, but David's London was yet a place of magic, a land of promise where fame and fortune await the bold.

Poor fool! let him wander in his paradise while he may.

(*To be continued.*)

